# A Comparative Study of the T Cell Stimulatory and Polarizing Capacity of Human Primary Blood Dendritic Cell Subsets

**DOI:** 10.1155/2016/3605643

**Published:** 2016-02-07

**Authors:** Simone P. Sittig, Ghaith Bakdash, Jorieke Weiden, Annette E. Sköld, Jurjen Tel, Carl G. Figdor, I. Jolanda M. de Vries, Gerty Schreibelt

**Affiliations:** ^1^Department of Tumor Immunology, Radboud University Medical Center, Radboud Institute for Molecular Life Sciences, 6500 HB Nijmegen, Netherlands; ^2^Department of Oncology-Pathology, Cancer Center Karolinska, Karolinska Institutet, 171 76 Stockholm, Sweden; ^3^Department of Medical Oncology, Radboud University Medical Center, Radboud Institute for Molecular Life Sciences, 6500 HB Nijmegen, Netherlands

## Abstract

Dendritic cells (DCs) are central players of immune responses; they become activated upon infection or inflammation and migrate to lymph nodes, where they can initiate an antigen-specific immune response by activating naive T cells. Two major types of naturally occurring DCs circulate in peripheral blood, namely, myeloid and plasmacytoid DCs (pDCs). Myeloid DCs (mDCs) can be subdivided based on the expression of either CD1c or CD141. These human DC subsets differ in surface marker expression, Toll-like receptor (TLR) repertoire, and transcriptional profile, suggesting functional differences between them. Here, we directly compared the capacity of human blood mDCs and pDCs to activate and polarize CD4^+^ T cells. CD141^+^ mDCs show an overall more mature phenotype over CD1c^+^ mDC and pDCs; they produce less IL-10 and more IL-12 than CD1c^+^ mDCs. Despite these differences, all subsets can induce the production of IFN-*γ* in naive CD4^+^ T cells. CD1c^+^ and CD141^+^ mDCs especially induce a strong T helper 1 profile. Importantly, naive CD4^+^ T cells are not polarized towards regulatory T cells by any subset. These findings further establish all three human blood DCs—despite their differences—as promising candidates for immunostimulatory effectors in cancer immunotherapy.

## 1. Introduction

Dendritic cells (DCs) are professional antigen-presenting cells that possess the unique capacity to activate and prime naive CD4^+^ and CD8^+^ T cells [1]. They form a heterogeneous population consisting of specialized DC subsets that differ in their surface marker expression, molecular phenotype, and antigen-processing and antigen-presentation capacity [2–4]. In peripheral blood, at least two major types of DCs can be distinguished, namely, myeloid DCs (mDCs) and plasmacytoid DCs (pDCs) [5, 6]. Myeloid DCs express high levels of CD11c and can further be subdivided based on the differential expression of either CD1c (blood dendritic cell antigen 1 = BDCA1) or CD141 (BDCA3). Each DC subset has its own repertoire of Toll-like receptors (TLRs), underlining their functional specialization [3, 7]. Plasmacytoid DCs express mainly TLR7 and TLR9. Both mDC subsets express TLR3 and TLR8 among others, although expression levels of TLR3 are much higher in CD141^+^ mDCs [7]. Plasmacytoid DCs are key effectors of innate immune responses due to their capacity to produce large amounts of type I IFNs in response to bacterial or viral infections; this production can also be induced by TLR agonists such as R848 and oligodeoxynucleotides class C (CpG) [8, 9]. Besides their role in the innate immune system, pDCs also participate in priming T helper (Th) cells, depending on the stimulus they receive (summarized in [9]). Myeloid DCs, on the other hand, have the capacity to produce the Th1 skewing cytokine interleukin- (IL-) 12. For both pDCs and mDCs, it has been shown that they induce proliferation in an allogeneic setting and that they can cross-present exogenous antigens to prime CD8^+^ T cells [10–16].

As a result of their unique capacity to orchestrate adaptive immune responses, DCs are being exploited for cancer immunotherapy. Recently, more advanced examination of primary blood DCs has come within reach through the availability of efficient isolation techniques. Primary DCs are hypothesized to be stronger inducers of anticancer responses than monocyte derived DCs in cell-based vaccination strategies since they differentiate* in vivo* and require only short* ex vivo* handling. The first clinical studies utilizing primary blood DCs have recently been conducted by our group, demonstrating the safety and efficacy of CD1c^+^ mDCs and pDCs in cancer immunotherapy [17, 18].

In order for DC-based immunotherapy to elicit potent antitumor T cell responses, the administered DCs need to raise an immune-stimulatory rather than tolerogenic T cell response [19]. Naive T cells will proliferate upon encounter with antigen-presenting cells presenting their specific antigen in the presence of costimulatory signals. The nature of costimulation and cytokines from the DC will influence the polarization of the T cells into different T helper phenotypes such as Th1, Th2, and Th17 or regulatory T cells (Tregs). For example, the presence of IL-12 promotes the induction of Th1 cells, whereas IL-10 inhibits induction of Th1 cells and promotes the differentiation of Tregs [20, 21]. In antiviral responses, Th1 cells and antigen-specific cytotoxic CD8^+^ T cells are elicited to eradicate cells infected by the virus. This type of immune response is also highly desirable in antitumor strategies, in which the aim is to eradicate tumor cells. Toll-like receptor ligands such as polyinosinic:polycytidylic acid (polyI:C), R848, and CpG have been shown to possess Th1 polarizing capacity when used as adjuvants or maturation agents for DCs [22–26].

To be able to successfully manipulate T cell responses for therapeutic strategies, a better understanding of the functional specialization of human DC subsets is needed. In this study, we compared the CD4^+^ T cell stimulatory and polarizing capacity of human blood mDCs and pDCs side by side—especially the capacity to induce Th1 responses upon differential stimulation.

## 2. Material and Methods

### 2.1. Cells

Human blood DCs were isolated from buffy coats (Sanquin) obtained from healthy volunteers after written informed consent and according to institutional guidelines. PBMCs were purified via Ficoll density gradient centrifugation (Lymphoprep by Axis-Shield). Monocytes were depleted via plastic adherence.

DCs were isolated by fluorescence-activated cell sorting (FACS). For this, lineage positive cells were depleted from PBMCs either with Dynabeads Human DC enrichment kit (Invitrogen by Life Technologies) or with anti-FITC microbeads (Miltenyi Biotec) after incubation with FITC-conjugated anti-Lin1 antibody cocktail (CD3^+^CD14^+^CD16^+^CD19^+^CD20^+^CD56^+^) (BD Biosciences). The remaining cells were labeled with FITC-conjugated anti-Lin1 antibody cocktail (BD Biosciences), PE-Cy7-conjugated anti-HLA-DR (BD Biosciences), BV421-conjugated anti-CD1c (Biolegend), APC-conjugated anti-CD141 (Miltenyi Biotec), and PE-conjugated anti-BDCA4 (Miltenyi Biotec). Subsets were sorted to obtain CD1c^+^ mDCs, CD141^+^ mDCs, or pDCs, respectively (purity 98–100%) (see Suppl. Fig. 1 in Supplementary Material available online at http://dx.doi.org/10.1155/2016/3605643). In some experiments, CD1c^+^ mDCs were isolated from PBMCs with a CD1c^+^ DC isolation kit (Miltenyi Biotec). CD141^+^ mDCs and pDCs were isolated from PBLs by positive selection with anti-CD141 (CD141) and anti-BDCA4 magnetic microbeads, respectively (Miltenyi Biotec). Purity was assessed by flow cytometry (85–97%). Naive CD4^+^ T cells were isolated from PBLs by depleting CD4^−^ cells with MACS MultiSort beads and additional use of PE-conjugated anti-CD45RO (Dako) and anti-PE beads (Miltenyi Biotec) for the depletion of CD45RO^+^ memory T cells (purity > 95%).

**Figure 1:**
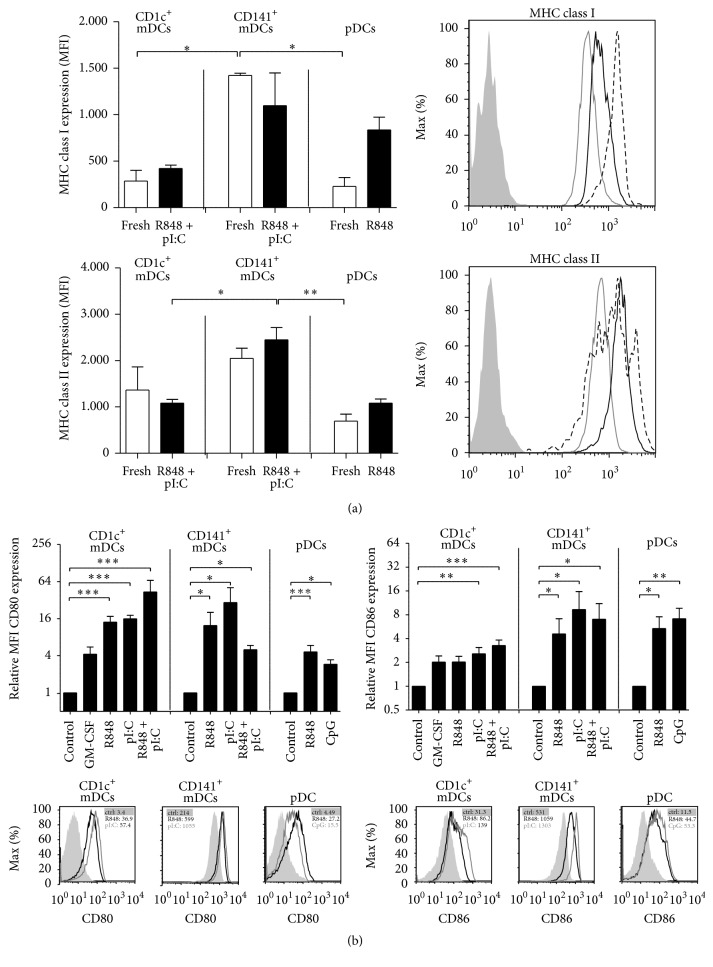
Phenotype of human blood DCs upon TLR stimulation. (a) MHC class I (HLA-ABC) and MHC class II (HLA-DR) expression was analyzed by flow cytometry of DCs kept at 4°C or DCs stimulated and cultured overnight. Myeloid DCs were stimulated with R848 and polyI:C (pI:C), whereas pDCs were stimulated with R848 alone. The bar graphs show the mean ± SEM of the mean fluorescent intensity (MFI) (*n* ≥ 3) and the histogram shows expression of freshly isolated DC subsets from a single representative donor (filled histogram: isotype control; grey line: pDCs, black line: CD1c^+^ mDCs, dashed line: CD141^+^ mDCs). (b) The bar graphs (upper panel) show the fold change ± SEM of the MFI for surface expression of the costimulatory molecules CD80 and CD86 after overnight stimulation with reference to cells cultured in medium alone (or IL-3 for pDCs) (*n* ≥ 4). The histograms (lower panel) show CD80 and CD86 expression from a single representative donor (insets: MFI values). Significance was determined by Kruskal-Wallis test followed by Dunn's testing comparing different conditions of the same subset (^*∗*^ *P* < .05; ^*∗∗*^ *P* < .01; ^*∗∗∗*^ *P* < .001).

**Figure 2:**
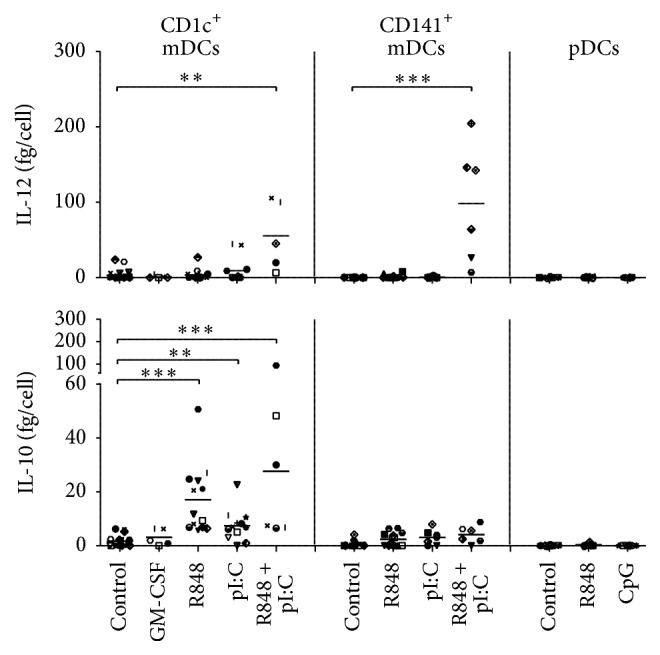
TLR ligation induces differential cytokine production by human blood DCs. The DCs were stimulated as indicated and cultured overnight at 37°C. IL-10 and IL-12p70 production was analyzed in supernatants of overnight cultures by standard sandwich ELISA (*n* ≥ 6). Each symbol represents one donor (also across the subsets). Significance was determined by Kruskal-Wallis test followed by Dunn's testing comparing different conditions of the same subset ( ^*∗∗*^ *P* < .01; ^*∗∗∗*^ *P* < .001).

**Figure 3:**
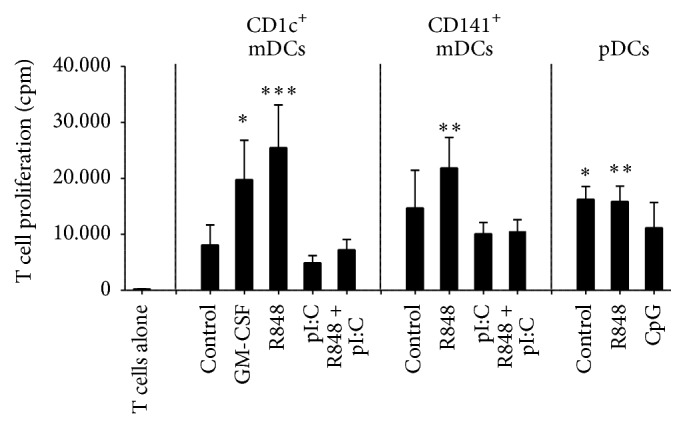
Human blood DCs induce proliferation of naive CD4^+^ T cells. Human CD1c^+^ mDCs, CD141^+^ mDCs, and pDCs were cultured overnight with the stimuli as indicated. The next day, allogeneic naive CD4^+^ T cells were added to the DCs (ratio 5 : 1). Proliferation was measured at day four of coculture by determining tritiated thymidine incorporation. The graph shows the mean proliferation ± SEM in counts per minute [cpm] (*n* ≥ 4). Each experiment was performed in triplicate for CD1c^+^ mDCs and pDCs and in duplicate for CD141^+^ mDCs. Significance was tested for each subset in comparison with T cells alone by Kruskal-Wallis test followed by Dunn's testing (^*∗*^ *P* < .05; ^*∗∗*^ *P* < .01; ^*∗∗∗*^ *P* < .001).

**Figure 4:**
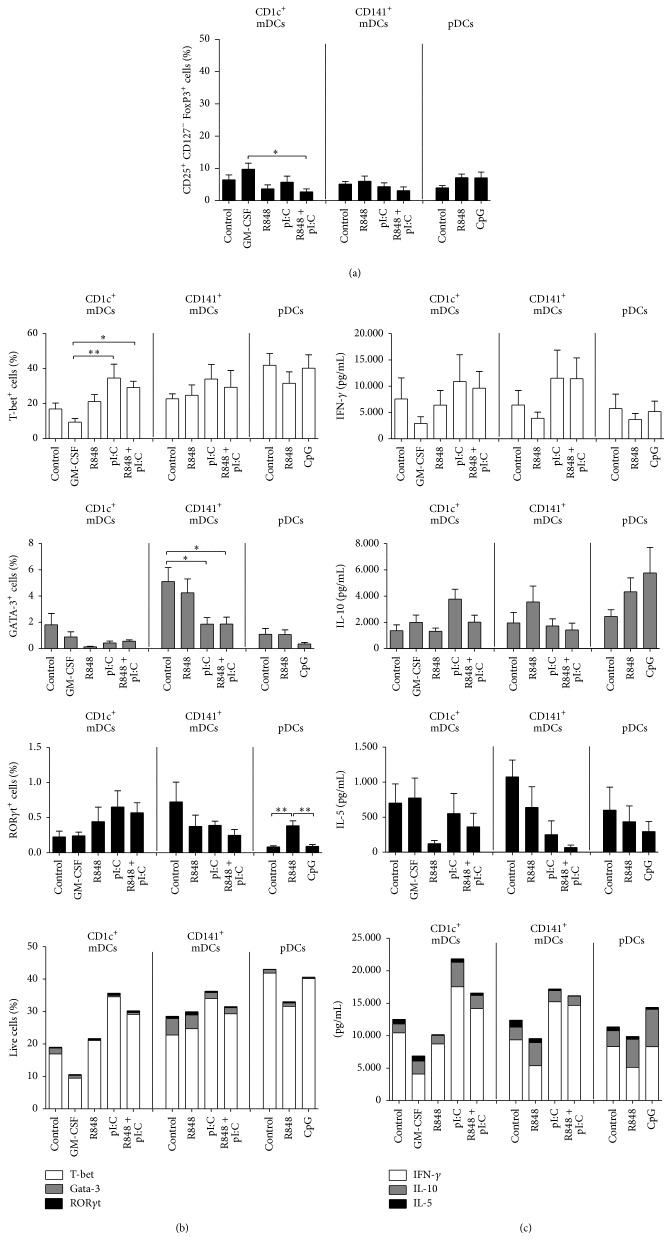
Mature human DC subsets can skew naive CD4^+^ T cells towards Th1 phenotype and do not induce a big population of Tregs. Human blood DCs were incubated with the indicated stimuli. The next day, allogeneic naive CD4^+^ T cells were added to the DCs together with a low concentration of the superantigen SEB (10 pg/mL) and cultured until resting (11–13 days). (a) These CD4^+^ T cells were analyzed by flow cytometry for presence of a Treg population (CD25^+^CD127^−^FoxP3^+^ CD4^+^ T cells) (*n* ≥ 5). (b) The cells were also stained for the expression of transcription factors T-bet, Gata-3, and ROR*γ*t. In the lower panel, all three transcription factors are depicted in a single bar graph (mean value for each). (c) Furthermore, 5 × 10^4^ of these CD4^+^ T cells were restimulated for 24 hrs with anti-CD3/anti-CD28 beads. Supernatants were analyzed for IL-5, IL-10, and IFN-*γ* by sandwich ELISA (*n* ≥ 4). The bar graphs show mean cytokine production ± SEM. In the lower panel, all three cytokines are depicted in a single bar graph (mean value for each cytokine). Significance comparing different conditions of the same subset was determined by Kruskal-Wallis test followed by Dunn's testing (a and c), by a 1-way ANOVA followed by Tukey testing or a paired *t*-test (b) (^*∗*^ *P* < .05; ^*∗∗*^ *P* < .01).

All cells were cultured in X-VIVO 15 medium (Lonza) supplemented with 2% human serum (Sigma-Aldrich). The DCs were stimulated with the following TLR ligands: 4 *μ*g/mL R848 (Axxora), 2 *μ*g/mL polyI:C (Sigma) (Figures 1 and 2) or 20 *μ*g/mL polyI:C (Enzo Life Sciences) (Figures 3 and 4), 450 U/mL GM-CSF (Cellgenix), or 5 *μ*g/mL CpG-C (designated CpG throughout text; Enzo Life Sciences). For the control condition of pDCs, the medium was supplemented with 10 ng/mL recombinant human IL-3 (Cellgenix) to ensure pDC survival.

Cell sorting was performed on a BD Aria and flow cytometry on a BD Calibur or BD Verse. The flow cytometry data was analyzed by FlowJo software.

### 2.2. Phenotype and Cytokine Production of TLR Activated DCs

The DC subsets were incubated overnight at 37°C with different stimuli in triplicate (CD1c^+^ mDCs, pDCs) or in duplicate (CD141^+^ mDCs). The next day, supernatants were taken and cells were labeled with PE-conjugated anti-MHC class I and FITC-conjugated anti-MHC class II (BD), PE-conjugated anti-CD80 (BD Biosciences), and PE-conjugated anti-CD86 (BD Biosciences). Marker expression was determined by flow cytometry (BD Calibur and FlowJo software). Supernatants were analyzed for IL-10 (eBioscience) and IL-12p70 (M122 and M121B by Pierce Endogen, standard by BD Biosciences) by standard sandwich ELISA. Depicted in Figure 2 is the cytokine production by 50,000 DCs in a volume of 100 *µ*L. For CD141^+^ mDCs, in some instances fewer cells were cultured. In all instances, cytokine production per cell was calculated.

### 2.3. T Cell Proliferation with Allogeneic Naive CD4^+^ T Cells

CD1c^+^ mDCs, CD141^+^ mDCs, or pDCs (1 × 10^4^ cells) were incubated overnight at 37°C with different stimuli in triplicate. The next day, allogeneic naive CD4^+^ T cells were added to the DCs at a ratio of 1 : 5 (DC : T cell). Proliferative responses were determined by adding 1 *μ*Ci [0.037 MBq]/well of tritiated thymidine (^3^H) (MP Biomedicals) to the cells after three days of coculture. ^3^H incorporation over a time course of 16 hours was measured with a scintillation counter.

### 2.4. Cocultures of DCs with Naive CD4^+^ T Cells and Analysis of the CD4^+^ T Cell Phenotype

Dendritic cells (1 × 10^4^) were stimulated overnight with the different stimuli in 100 *μ*L medium. Next, allogeneic naive CD4^+^ T cells (4 × 10^4^) were added at a ratio of 1 : 4 (DC : T cell) in a final volume of 200 *µ*L medium containing 10 pg/mL superantigen* Staphylococcus aureus* enterotoxin B (SEB) (Sigma). At day 5, human rIL-2 (20 IU/mL, Novartis) was added and the cultures expanded for the next 6–8 days. On days 11–13, resting T cells were counted and analyzed by flow cytometry with three panels. Panel 1 includes anti-CD25-APC (BD Bioscience), anti-CD127-PE (eBioscience), and anti-Foxp3-A488 (eBioscience). Panel 2 includes anti-T-bet-A488, anti-GATA-3-PE, and anti-ROR*γ*t-APC (all eBioscience). Panel 3 includes anti-CD45RO-APC (BD Bioscience), anti-CD197 (R&D Systems) with goat-anti-mouse IgG2a-A488 (Life Technologies), and anti-CD62-L (eBioscience) with rat-anti-mouse IgG1-PE (BD Pharmingen). The population of Tregs was determined by selecting CD25^+^ CD127^−^ cells and subsequently gating on the FoxP3^+^ population (Suppl. Fig. 2a). From CD45RO^+^ cells, T_CM_ were determined by further gating on CD197^+^/CD62-L^+^ and T_EM_ were determined by further gating on CD197^−^ cells. Both populations are shown as percentage of live cells (forward-sideward scatter) (Suppl. Fig. 2b).

Furthermore, 5 × 10^4^ of the T cells of each condition were restimulated with 5 × 10^4^ anti-CD3/anti-CD28 beads (Dynabeads Gibco by Life Technologies) in triplicate and supernatants from 24-hour cultures were analyzed for levels of IFN-*γ* (Pierce Endogen), IL-5, and IL-10 (eBioscience) by standard sandwich ELISA.

### 2.5. Statistical Analysis

Data were analyzed by Kruskal-Wallis test followed by Dunn's testing, by a 1-way ANOVA followed by Tukey testing or with paired Student's *t*-test using Prism5 (GraphPad Prism5). Statistical significance was defined as <0.05 (^*∗*^ *P* < .05; ^*∗∗*^ *P* < .01; ^*∗∗∗*^ *P* < .001).

## 3. Results

### 3.1. TLR Ligation Increases Expression of Costimulatory Molecules by Human Blood DCs

High expression of MHC molecules is a hallmark of DCs, underlining their antigen-presenting capacities. Accordingly, we found high levels of both MHC class I and MHC class II molecules on all three DC subsets (Figure 1(a)). The levels of both MHC class I and MHC class II molecules were highest for CD141^+^ mDCs and comparable for CD1c^+^ mDCs and pDCs, both on freshly isolated cells and after TLR activation.

The expression of costimulatory molecules by DCs is essential to activate T cells and can be induced by TLR ligands. Throughout the study, CD1c^+^ and CD141^+^ mDC maturation was achieved by polyI:C, R848, or a combination of both. CD1c^+^ mDCs were also stimulated with granulocyte-macrophage colony-stimulating factor (GM-CSF). Plasmacytoid DCs were stimulated with R848 or CpG and IL-3 used for the control to secure pDC survival. On CD1c^+^ mDCs, the costimulatory molecule CD86 was already highly expressed after overnight culture in medium alone; on CD141^+^ mDCs, this holds true for the expression of both CD80 and CD86 (Figure 1(b)). In comparison, CD141^+^ mDCs showed the highest expression of CD80 and CD86, both after culturing in medium alone or after TLR ligation (Figure 1(b)). Although CD80 and CD86 molecules were expressed already at high levels on immature DCs, expression of both molecules was significantly increased upon culture with TLR ligands on all DCs.

### 3.2. TLR Ligation Induces Differential Cytokine Production by Human Blood DCs

Dendritic cell-derived IL-10 is known to inhibit Th1 cells and induce type 1 Tregs (Tregs producing IL-10), whereas IL-12 is a Th1-inducing cytokine and therefore desirable in the context of anticancer therapy [20, 21]. We directly compared the secretion of these cytokines by the differentially stimulated DC subsets. Plasmacytoid DCs did not secrete IL-10 or IL-12 at detectable levels, whereas CD1c^+^ and CD141^+^ mDCs secreted both IL-10 and IL-12 at differential levels depending on the stimulus (Figure 2). R848 and polyI:C, alone or in combination, induced IL-10 production in CD1c^+^ mDCs, while only the combination of both TLR ligands induced a significant increase in the secretion of IL-12. CD141^+^ mDCs secreted low amounts of IL-10, irrespective of the stimulus and at lower levels than CD1c^+^ mDCs. We observed a significant increase in IL-12 production by CD141^+^ mDCs after stimulation with both polyI:C and R848, which is higher than the production by CD1c^+^ mDCs. We can therefore conclude that CD141^+^ mDCs produce less IL-10 and more IL-12 than CD1c^+^ mDCs.

### 3.3. Human Blood DCs Induce Proliferation of Allogeneic Naive CD4^+^ T Cells

A primary immune response constitutes the activation of naive T cells in response to antigen and their subsequent proliferation and differentiation. Besides recognition of their cognate antigen, naive T cells depend on costimulation by the antigen-presenting cell to start such a primary response. The ability of blood DCs to induce proliferation of naive T cells was directly compared by coculturing overnight stimulated pDCs and CD1c^+^ and CD141^+^ mDCs of the same donors with allogeneic naive CD4^+^ T cells. Proliferation was measured at day four by tritiated thymidine incorporation. All primary blood DC subsets showed the ability to induce proliferation of naive CD4^+^ T cells (Figure 3). Even so, R848-matured mDCs induced the highest levels of proliferation, while polyI:C maturation did not further increase proliferation as compared to unstimulated mDCs. For pDCs, R848 and IL-3 (control) treatment stimulate similar levels of naive CD4^+^ T cell proliferation, while the levels for CpG-treated pDCs tend to be lower than for R848 or IL-3.

Besides providing effector T cells, a primary immune response can generate immunological memory in the form of memory T cells. While central memory T cells (T_CM_) are responsible for rapid clonal expansion after reexposure to antigen and localize in lymphoid organs, effector memory T cells (T_EM_) localize in mucosal tissue and mediate rapid effector functions there. Although the formation and longevity of such memory cells can only be accurately measured* in vivo*, we wanted to get an idea of the individual capacities of the different DC subsets to induce them. For this, we cocultured naive, allogeneic CD4^+^ T cells with the differentially activated blood DC subsets until the T cells had ceased to proliferate [27]. At this resting state (after ~12 days), we analyzed their CD45RO, CCR7 (CD197), and L-selectin (CD62L) expression. The percentages of CD45RO^+^ CCR7^−^ (T_EM_) and CD45RO^+^ L-selectin^+^ CCR7^+^ (T_CM_) among the T cells did not differ significantly between the subsets or different stimuli (Suppl. Fig. 3 and 2b). At the time point measured, the T cells comprise a larger proportion of T_EM_ (mean 47.14%–71.51%) than T_CM_ (mean 13.47%–24%).

Taken together, all subsets can effectively induce proliferation of naive T cells and are probably able to induce memory T cells. However, mDCs induce significantly higher proliferation when matured with R848 in comparison to polyI:C maturation or culturing alone.

### 3.4. All Human Blood DCs Can Drive IFN-*γ* Production by Naive CD4^+^ T Cells and Do Not Induce Tregs

Dendritic cells play a critical role in the polarization of naive CD4^+^ T cells into different T helper phenotypes or Tregs. In a Th1 response, cytotoxic CD8^+^ T cells that are able to kill cells bearing their specific antigen are elicited. Therefore, this type of immune response is highly desirable in antitumor strategies. To compare the differential T cell stimulatory and polarizing capacity—especially the capacity to induce Th1 responses upon differential stimulation with polyI:C, R848, and CpG—and possible Treg induction by human blood mDCs and pDCs, naive CD4^+^ T cells were cocultured with the DC subsets until they reached resting state. Importantly, analysis of the resting T cells did not show a large fraction of Tregs for any DC subset or condition (mean 3%–10%) (Figure 4(a); Suppl. Fig. 2a). Although the differences are small, it is interesting to note that the percentages of these cells were lowest for polyI:C and R848-matured mDCs and were highest for GM-CSF-stimulated CD1c^+^ mDCs (mean 3% and 10%, resp.). For the pDC cocultures, there is a tendency of a higher proportion of Tregs being induced after R848 or CpG stimulation compared to the control (IL-3-treated cells) (mean 7%, 7%, and 4%, resp.). Furthermore, we analyzed the induction of Th subset-specifying transcription factors T-bet, GATA-3, and ROR*γ*t (Figure 4(b)). We found pronounced populations expressing T-bet across the subsets and stimuli (CD1c^+^ mDCs: 9%–35%, CD141^+^ mDCs: 23%–35%, and pDCs: 32%–42%), indicating Th1 polarization by all subsets. GATA-3 expression was overall low, indicating little Th2 polarization. CD141^+^ mDCs showed the most pronounced GATA-3 expression for control and R848 stimulation (mean 5.1% and 4.25%, resp.), which was significantly reduced with polyI:C or combined R848 and polyI:C stimulation of CD141^+^ mDCs (mean 1.87% for both). Furthermore, ROR*γ*t expression was only detected in a very small population of CD4^+^ T cells across the subsets (0.08%–0.72%), indicating Th1 rather than Th17 polarization of these cells.

Resting CD4^+^ T cells were also restimulated with anti-CD3/anti-CD28 beads and their supernatants analyzed for IL-5, IL-10, IL-17, and IFN-*γ* production to determine the Th1 polarization capacity of the DC subsets. Interleukin-5 is a Th2 cytokine, while IL-10 inhibits Th1 polarization and IFN-*γ* is a strong Th1 inducer [20, 21, 28]. Notably, coculture with all three blood DC subsets induced T cells with prominent IFN-*γ* production after restimulation even without TLR maturation (Figure 4(c)). T cells primed by CD141^+^ or CD1c^+^ mDCs induced prominent populations of T-bet expressing cells and secreted high levels of IFN-*γ*, indicating Th1 skewing (Figure 4(c), lower panel). GM-CSF-stimulated CD1c^+^ mDCs induced smaller populations of T-bet expressing cells and lower levels of IFN-*γ* and similar levels of both IL-5 and IL-10 as the medium control or TLR-matured DCs; therefore, GM-CSF maturation of CD1c^+^ mDCs does not induce the most potent Th1 response. Also pDCs induce a prominent population of T-bet expressing cells and IFN-*γ* release from restimulated CD4^+^ T cells, although the levels of IFN-*γ* are lower than for optimally stimulated mDCs. Plasmacytoid DCs induce similar levels of IL-5 in cocultured T cells as mDCs. However, the levels of IL-10 are higher, especially for R848 and CpG stimulated pDCs, which coincides with a tendency for a bigger proportion of Tregs (CD25^+^ CD127^−^ FoxP3^+^) induced in these conditions. We measured no IL-17 for pDCs and modest levels for mDCs stimulated with R848 or CD1c^+^ mDCs stimulated with the combination of R848 and polyI:C (Suppl. Fig. 4). Together with the ROR*γ*t expression data we conclude a Th1 rather than Th17 polarization of the naïve CD4^+^ T cells. In sum, all subsets polarize naive CD4^+^ T cells mainly towards Th1 cells with a strong T-bet signature producing mainly IFN-*γ* after restimulation.

## 4. Discussion

In order to manipulate T cell responses for DC-based cancer immunotherapy, a better understanding of the functional specialization of human DC subsets is needed. In this study, we compared the capacities of primary human blood mDCs and pDCs to activate and polarize CD4^+^ T cells side by side. We report that CD1c^+^ mDCs, CD141^+^ mDCs, and pDCs all induce proliferation of naive CD4^+^ T cells. Importantly, naive CD4^+^ T cells are not skewed towards a regulatory phenotype by coculture with either mature mDCs or pDCs. Despite differences in activation and cytokine profile, both CD141^+^ and CD1c^+^ mDCs polarize naive CD4^+^ T cells towards T cells with a strong IFN-*γ* signature; also pDCs induce IFN-*γ*, although at lower levels and accompanied by a higher IL-10 production.

While all DC subsets mature upon TLR ligation, we observed distinct cytokine responses for different subsets and stimuli. CD1c^+^ mDCs produced only a limited amount of IL-12 after maturation with either R848 or polyI:C alone, but production was significantly increased with a combination of these TLR ligands. Even higher levels of IL-12 are produced by CD141^+^ mDCs when stimulated with the combination of polyI:C and R848. In contrast to our findings, Nizzoli et al. did not find IL-12 production for CD141^+^ mDCs after combined polyI:C and R848 stimulation [29]. Other studies have shown that, in order to induce strong IL-12 responses in human and mouse DCs, both an innate trigger such as TLR ligation and a second trigger like ligation of CD40 by CD40L on T cells are needed [7, 30]. More recently, it has been shown for CD1c^+^ mDCs that the combination of the TLR ligands R848 and LPS can trigger significant IL-12 production [29]. In the case of CD141^+^ mDCs, a cocktail of polyI:C together with the cytokines IFN-*γ*, TNF-*α*, IFN-*α*, and IL-1*β* was shown to induce significant levels of IL-12 [31]. Hémont et al. showed that CD141^+^ mDCs produced less IL-12 as compared to CD1c^+^ mDCs for single TLR ligation but that a higher proportion produced IL-12 after TLR1/2 or TLR3 ligation in a whole blood assay [7]. Our data supports the notion that a single stimulus is not sufficient to induce high IL-12 production and with polyI:C and R848 we describe a new combination that can trigger substantial IL-12 secretion by both human mDC subsets.

All DC subsets induced proliferation of naive CD4^+^ T cells—regardless of the stimulus. The level of T cell proliferation induced by polyI:C-matured mDCs is similar to nonstimulated mDCs. Strikingly, GM-CSF-stimulated CD1c^+^ mDCs and R848-matured CD1c^+^ and CD141^+^ mDCs cause an extra boost in proliferation of naive CD4^+^ T cells. This is in accordance with an earlier study by Jongbloed et al., which described equally high induction of proliferation of naive CD4^+^ T cells for nonstimulated or polyI:C stimulated CD1c^+^ and CD141^+^ mDCs after six days [31]. Because of the upregulation of the expression of costimulatory molecules with both stimuli compared to untreated DCs, one would expect a higher proliferation rate than with untreated DCs. Certainly, other cytokines and immunostimulatory, but also immunoinhibitory, molecules expressed by cultured DCs are integrated into a single response by the T cells and possible differences in these factors might cause the observed differences in T cell proliferation.

Only a minor percentage of CD4^+^ T cells that grew out of cocultures with the different DC subsets displayed a Treg phenotype. Earlier studies suggest that pDCs can act as Th1, Th2, Th17, or even Treg inducers in T cell priming, depending on the stimulus they receive (summarized in [9]). One stimulus that can induce this regulatory T cell phenotype is CpG and the proposed mechanism is via the expression of inducible costimulator ligand (ICOS-L) [32]. Ito et al. show in their study that pDCs upregulate ICOS-L upon CpG maturation, which triggers IL-10 production of T cells but no production of IL-4, IL-5, or IL-13. This is in accordance with our findings, where we observed higher levels of IL-10 and a tendency of a higher proportion of Tregs for pDCs matured with CpG or R848 compared to matured mDCs but no elevated levels of IL-5. However, regardless of the stimulus we also found a strong T-bet expression and IFN-*γ* production by CD4^+^ T cells that had grown out of cocultures. Plasmacytoid DCs secrete large amounts of type I IFNs in response to bacterial or viral stimuli, including R848 and CpG [8]. Type I IFNs not only are important in innate responses, but can also help to skew T cells towards a Th1 phenotype [33]. Type I IFNs secreted by pDCs might play a role in the observed IFN-*γ* induction.

Regulatory T cell induction with functional effects on T cells has been described in one study for tissue mDCs of the skin [34]. We show here that primary blood mDCs induce only a low proportion of Tregs and, importantly, the overall CD4^+^ T cell population displays a Th1 phenotype after coculture (pronounced T-bet expression and high IFN-*γ* production) and no Th2 or Th17 phenotype. Myeloid DCs induced a strong Th1 phenotype in CD4^+^ T cells. This is in line with the ability of mDCs to produce IL-12 after combined polyI:C and R848 stimulation. For the other conditions, one can speculate whether the addition of the CD4^+^ T cells and therefore ligation of CD40 on the DCs give the needed second signal for IL-12 production and help the Th1 skewing. Different groups have shown that blood mDCs can induce IFN-*γ* production in naive CD4^+^ and CD8^+^ T cells [7, 35, 36]. Jongbloed et al. found that CD141^+^ mDCs were more potent than CD1c^+^ DCs at inducing IFN-*γ* responses in total CD4^+^ T cells, especially after polyI:C stimulation [31]. We found that although CD1c^+^ mDCs show a less mature phenotype than CD141^+^ mDCs including higher IL-10 and lower IL-12 production, CD1c^+^ and CD141^+^ mDCs induce similar IFN-*γ* responses after coculture with naive CD4^+^ T cells. However, there is a tendency for CD141^+^ mDCs to induce less IL-5 and IL-10 than CD1c^+^, also arguing for an overall stronger Th1 skewing by this subset. For CD141^+^ mDCs, high TLR3 expression and the ability to produce IFN-*λ* and CXCL10, both known to induce antiviral responses, all suggest their capability to induce Th1 skewing in T cells [7, 37, 38]. Certainly, R848 and polyI:C can trigger distinct pathways as TLR3 signals through a TRIF-to-IRF3 pathway, rather than an MyD88-to-IRF7 pathway that is used by TLR8. It is interesting to note that both mDC subsets react strongly and in a similar way to polyI:C, although the expression levels of TLR3 are much higher in CD141^+^ mDCs than in CD1c^+^ mDCs [7]. Likely, other receptors for polyI:C contribute to the response in one or both of the mDC subsets. The synthetic dsRNA analog is a ligand for multiple pathogen recognition receptors, and besides TLR3 also triggers cytosolic RIG-I-like receptors that are expressed by mDCs [39, 40]. Perrot et al. suggest in a study on mDCs and NK cells that both TLR3 stimulation and RIG-I-like receptor ligation are needed for IFN-*γ* induction by mDCs [41].

In addition to their CD4^+^ T cell activating capacities, all three subsets can cross-present exogenous antigens for cognate restimulation of previously activated CD8^+^ T cells [10–14], making them promising candidates for DC-based vaccination strategies against cancer. Both CD1c^+^ mDCs and pDCs have generated promising results in first clinical studies utilizing these primary blood DC subsets as vaccines [17, 18]. These studies support their excellent* in vivo* functioning and mark them as the next generation of cancer vaccines. In this context, we have learned from the current work that GM-CSF is not the optimal stimulus for CD1c^+^ mDCs, since GM-CSF stimulation showed an overall lower Th1 skewing capacity and induced more Tregs than other stimuli. While maturation with polyI:C or the combination of polyI:C and R848 induces the most pronounced Th1 skewing, the number of T cells that grow out with these stimuli is lower than, for example, with R848 stimulation. Considering the proliferation data and the similar polarization capacity by all subsets and with all stimuli including control DCs, one can only speculate about a recommendation for a suited stimulation of DCs for DC-based vaccination strategies. However, TLR maturation probably has extra benefits beyond skewing T cell polarization. For example, TLR activation of DCs can lead to the upregulation of otherwise unexpressed TLR ligands in the DCs [42], making them sensitive to a broader range of danger signals. Furthermore, in an* in vivo* situation also other cell types might play a crucial role for the overall outcome of a therapy. Such cells include NK and CD8^+^ T cells, for which type I IFNs and IL-12—secreted at higher levels upon TLR maturation—are important [43–46]. As discussed above, CD141^+^ mDCs certainly display promising properties for DC-based anticancer vaccination strategies. Besides their Th1-inducing capacity, human CD141^+^ mDCs are also excellent cross-presenters of exogenous antigens to CD8^+^ T cells. While some publications show superior cross-presentation capacity of CD141^+^ mDCs [31, 47–49] and put them forward as the human counterparts of mouse CD8*α* ^+^ DCs [31, 47–50], other studies suggest that the different human DCs subsets bear similar cross-presentation capacities at least for soluble antigens delivered through early endosomes [14, 15, 51]. The type and size of the antigen as well as the compartments they are targeted to probably underlie these differing outcomes (reviewed in [52, 53]). In addition to using single subsets for therapeutic approaches, we hypothesize that using a combination of several DC subsets could further increase T cell activating properties, since earlier studies have shown that cell-cell interactions as well as soluble factors can act to cross-activate the different DCs (summarized in [54]). With this comparative study, we have reinforced the establishment of human circulating CD1c^+^ mDCs, CD141^+^ mDCs, and pDCs as promising candidates for DC-based immunotherapy in the context of cancer.

## Supplementary Material

Supplementary Figure 1: Gating strategy for the sorting of primary blood DC subsets and purity. Before fluorescence-activated cell sorting (FACS), lineage (CD3, CD14, CD16, CD19, CD20, CD56) positive cells were depleted from PBMCs. The remaining cells were incubated with the antibodies recognizing lineage markers (FITC), HLA-DR (PE-C7), CD1c (BV421), CD141 (APC), and BDCA4 (PE). (a) The cells were sorted from lineage nega-tive, HLR-DR^+/high^ cells into three different populations, based on the expres-sion of CD1c, CD141 or BDCA4 to obtain CD1c^+^ mDCs, CD141^+^ mDCs or pDCs, respectively. (b) Purity of isolated cells was assessed by gating on life cells and analyzing expression of HLA-DR and exclusive expression of CD1c, CD141 or BDCA4 (not shown). Shown is HLA-DR with either CD1c, CD141 or BDCA4.
Supplementary Figure 2: Gating strategy for regulatory T cells and effector memory phenotype. (a) The population of regulatory T cells was determined by selecting CD25^+^ CD127^−^ cells and subsequently gating on the FoxP3^+^ population. The populations are shown as percentage of live cells in figure 4a. Dead cells were excluded on the basis of the forward-sideward scatter. (b) Central and effector memory T cells were determined on the basis of surface staining of CD45RO (APC), CD197 (CCR7) (+ A488-conjugated secondary Ab) and CD62-L (L-selectin) (+ PE-conjugated secondary Ab). From CD45RO+ cells, central memory T cells (T_CM_) were determined by further gating on CCR7^+^/L-selectin^+^ and effector memory T cells (T_EM_) were determined by further gating on CCR7^−^ cells; both populations are shown as percentage of live cells in supplementary figure 3.
Supplementary Figure 3: Human DC subsets induce an effector memory pheno-type in naive CD4+ T cells Human blood DCs were incubated with the indicated stimuli. The next day, allogeneic naive CD4 ^+^ T cells were added to the DCs together with a low concentration of the superantigen SEB (10 pg/ml) and cultured until resting (11-13 days). The memory phenotype (n=5) was investigated using flow cytometry. The bar graphs show the mean percentage ± SEM of effector (a) and central (b) memory CD4 ^+^ T cells gated from live cells (TEM: CD45RO ^+^ CCR7 ^−^ and TCM: CD45RO^+^ CCR7 ^+^ L-selectin ^+^). Significance was determined by Kruskal-Wallis test followed by Dunns testing comparing the different conditions of the same subset.
Supplementary Figure 4: IL-17 production of re-stimulated CD4^+^ T cells after co-culture with the DCs Human blood DCs were incubated with the indicated stimuli. The next day, allogeneic naive CD4 ^+^ T cells were added to the DCs together with a low concentration of the superantigen SEB (10 pg/ml) and cultured until resting (11-13 days). These CD4^+^ T cells were re-stimulated for 24 hrs with anti-CD3/CD28-beads. Supernatants were analyzed for IL-17 by sandwich ELISA (n=6 for CD1c^+^ mDCs and pDCs; n=1-4 for CD141^+^ mDCs). The graph shows mean cytokine production. Each symbol represents one donor (also across the subsets).

## Abbreviations

mDC: Myeloid dendritic cell
pDC: Plasmacytoid dendritic cell
BDCA: Blood DC antigen
CpG: Oligodeoxynucleotides class C
polyI:C: Polyinosinic:polycytidylic acid
T_CM_: Central memory T cell
T_EM_: Effector memory T cell.
