# Supplementary material for: A Comparative Study of the T Cell Stimulatory and Polarizing Capacity of Human Primary Blood Dendritic Cell Subsets
Source: Mediators Inflamm. 2016 Feb 7;2016:3605643. doi: 10.1155/2016/3605643 (PMC4761397; doi:10.1155/2016/3605643)
Supplement: Supplementary file 1 — Supplementary Figure 1: Gating strategy for the sorting of primary blood DC subsets and purity. Before fluorescence-activated cell sorting (FACS), lineage (CD3, CD14, CD16, CD19, CD20, CD56) positive cells were depleted from PBMCs. The remaining cells were incubated with the antibodies recognizing lineage markers (FITC), HLA-DR (PE-C7), CD1c (BV421), CD141 (APC), and BDCA4 (PE). (a) The cells were sorted from lineage nega-tive, HLR-DR+/high cells into three different populations, based on the expres-sion of CD1c, CD141 or BDCA4 to obtain CD1c+ mDCs, CD141+ mDCs or pDCs, respectively. (b) Purity of isolated cells was assessed by gating on life cells and analyzing expression of HLA-DR and exclusive expression of CD1c, CD141 or BDCA4 (not shown). Shown is HLA-DR with either CD1c, CD141 or BDCA4. Supplementary Figure 2: Gating strategy for regulatory T cells and effector memory phenotype. (a) The population of regulatory T cells was determined by selecting CD25+ CD127− cells and subsequently gating on the FoxP3+ population. The populations are shown as percentage of live cells in figure 4a. Dead cells were excluded on the basis of the forward-sideward scatter. (b) Central and effector memory T cells were determined on the basis of surface staining of CD45RO (APC), CD197 (CCR7) (+ A488-conjugated secondary Ab) and CD62-L (L-selectin) (+ PE-conjugated secondary Ab). From CD45RO+ cells, central memory T cells (TCM) were determined by further gating on CCR7+/L-selectin+ and effector memory T cells (TEM) were determined by further gating on CCR7− cells; both populations are shown as percentage of live cells in supplementary figure 3. Supplementary Figure 3: Human DC subsets induce an effector memory pheno-type in naive CD4+ T cells Human blood DCs were incubated with the indicated stimuli. The next day, allogeneic naive CD4 + T cells were added to the DCs together with a low concentration of the superantigen SEB (10 pg/ml) and cultured until resting (11-13 days). The memory [file 3605643.f1.pdf]

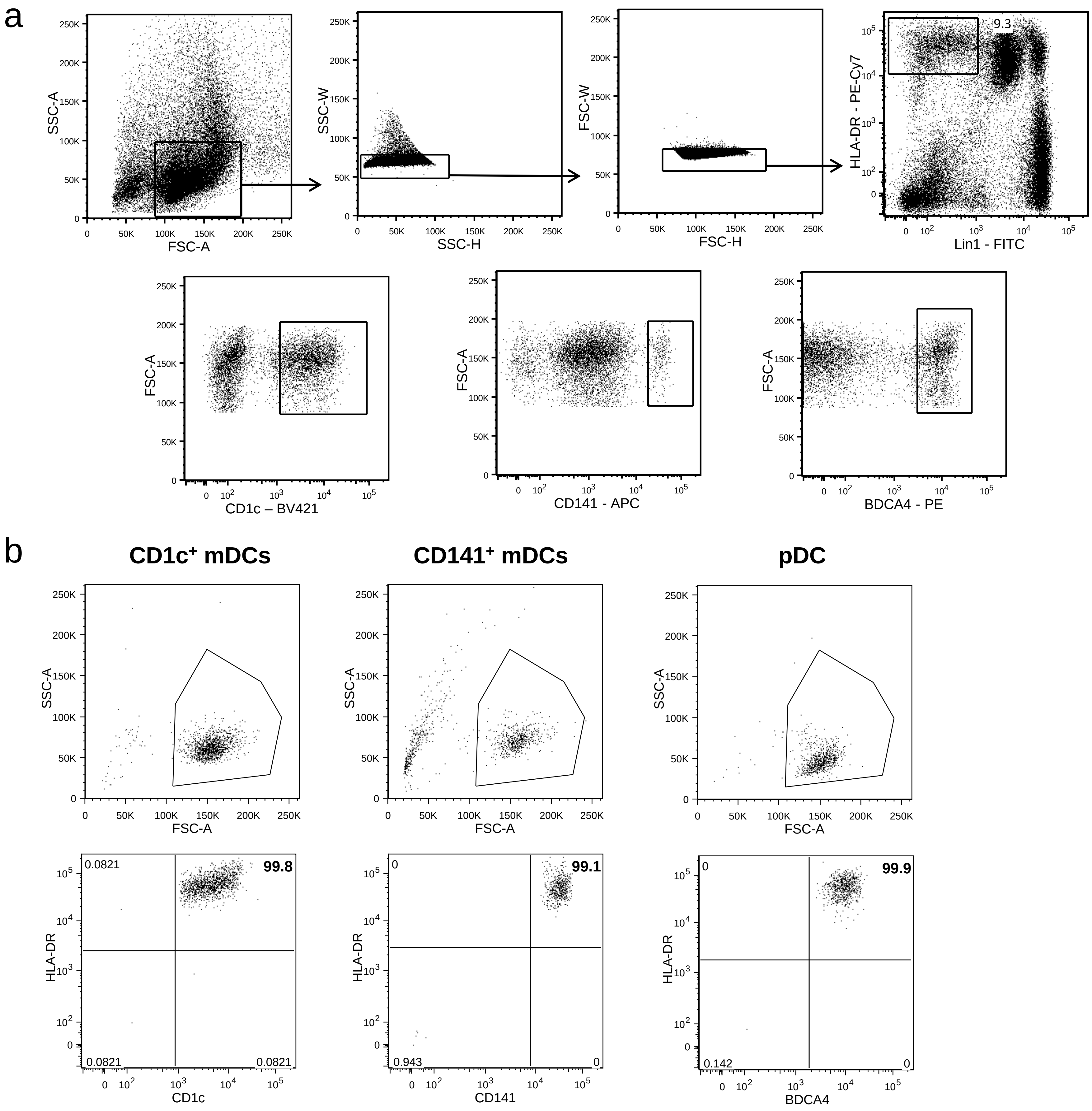

Supplementary Figure 1: **Gating strategy for the sorting of primary blood DC subsets and purity.** Before fluorescence-activated cell sorting (FACS), lineage (CD3, CD14, CD16, CD19, CD20, CD56) positive cells were depleted from PBMCs. The remaining cells were incubated with the antibodies recognizing lineage markers (FITC), HLA-DR (PE-C7), CD1c (BV421), CD141 (APC), and BDCA4 (PE). (**a**) The cells were sorted from lineage negative, HLR-DR$^{+/high}$ cells into three different populations, based on the expression of CD1c, CD141 or BDCA4 to obtain CD1c^{+} mDCs, CD141^{+} mDCs or pDCs, respectively. (**b**) Purity of isolated cells was assessed by gating on life cells and analyzing expression of HLA-DR and exclusive expression of CD1c, CD141 or BDCA4 (not shown). Shown is HLA-DR with either CD1c, CD141 or BDCA4.

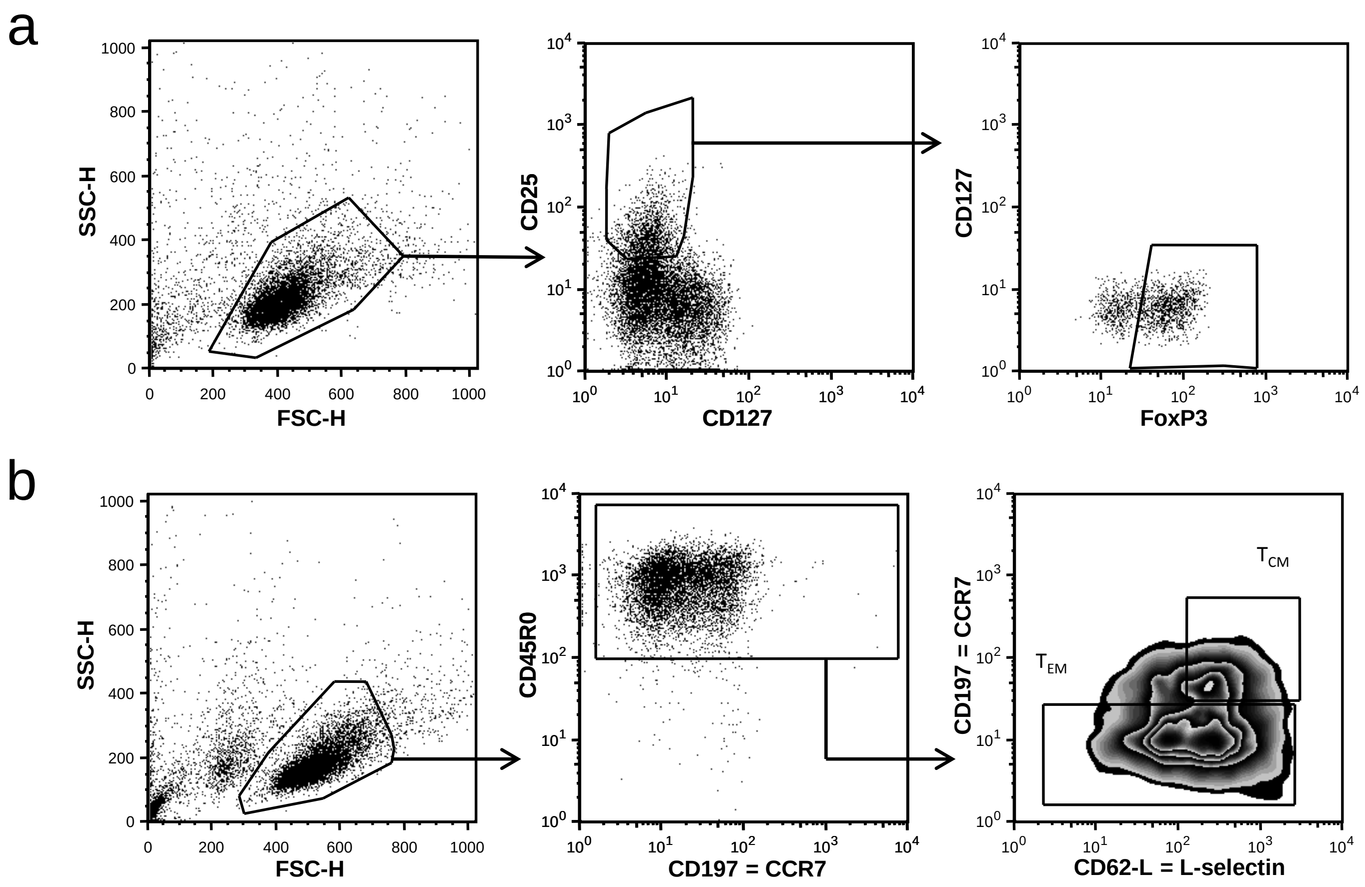

Supplementary Figure 2: Gating strategy for regulatory T cells and effector memory phenotype. (**a**) The population of regulatory T cells was determined by selecting $CD25^{+}$ $CD127^{-}$ cells and subsequently gating on the $FoxP3^{+}$ population. The populations are shown as percentage of live cells in figure 4a. Dead cells were excluded on the basis of the forward-sideward scatter. (**b**) Central and effector memory T cells were determined on the basis of surface staining of CD45RO (APC), CD197 (CCR7) (+ A488-conjugated secondary Ab) and CD62-L (L-selectin) (+ PE-conjugated secondary Ab). From $CD45RO^{+}$ cells, central memory T cells (T_{CM}) were determined by further gating on $CCR7^{+}$/L-$selectin^{+}$ and effector memory T cells (T_{EM}) were determined by further gating on $CCR7^{-}$ cells; both populations are shown as percentage of live cells in supplementary figure 3.

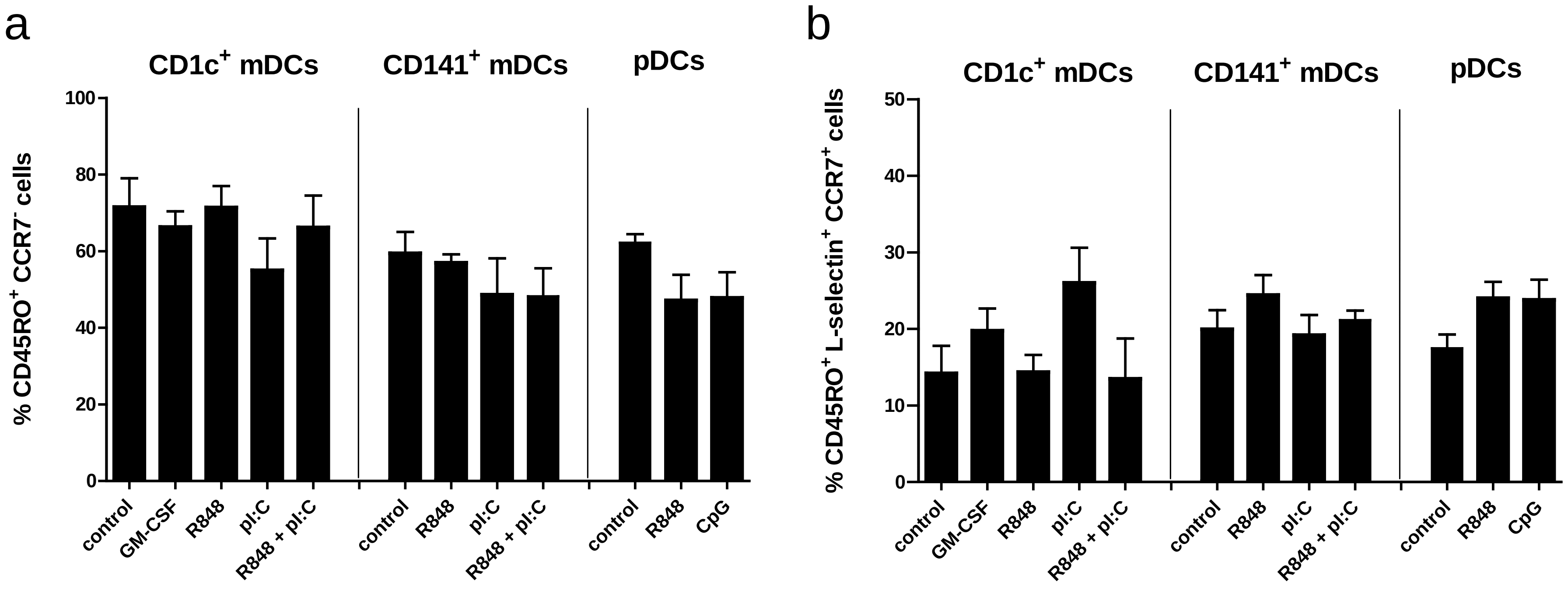

Supplementary Figure 3: **Human DC subsets induce an effector memory phenotype in naive CD4^{+} T cells** Human blood DCs were incubated with the indicated stimuli. The next day, allogeneic naive CD4^{+} T cells were added to the DCs together with a low concentration of the superantigen SEB (10 pg/ml) and cultured until resting (11-13 days). The memory phenotype (n=5) was investigated using flow cytometry. The bar graphs show the mean percentage ± SEM of effector (a) and central (b) memory CD4^{+} T cells gated from live cells (TEM: CD45RO^{+} CCR7^{-} and TCM: CD45RO^{+} CCR7^{+} L-selectin^{+}). Significance was determined by Kruskal-Wallis test followed by Dunns testing comparing the different conditions of the same subset.

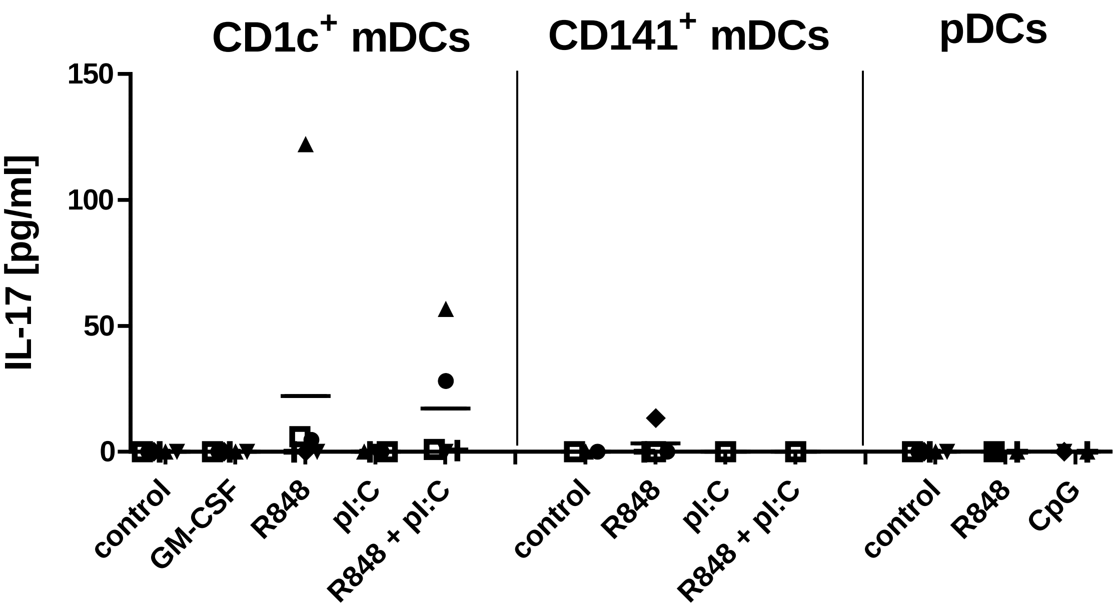

Supplementary Figure 4: IL-17 production of re-stimulated CD4+ T cells after co-culture with the DCs Human blood DCs were incubated with the indicated stimuli. The next day, allogeneic naive CD4+ T cells were added to the DCs together with a low concentration of the superantigen SEB (10 pg/ml) and cultured until resting (11-13 days). These CD4+ T cells were re-stimulated for 24 hrs with anti-CD3/CD28-beads. Supernatants were analyzed for IL-17 by sandwich ELISA (n=6 for CD1c+ mDCs and pDCs; n=1-4 for CD141+ mDCs). The graph shows mean cytokine production. Each symbol represents one donor (also across the subsets).
